# Take my breath away—mitochondrial dysfunction drives CD8^+^ T cell exhaustion

**DOI:** 10.1038/s41435-023-00233-8

**Published:** 2024-01-22

**Authors:** Felix Clemens Richter, Mariia Saliutina, Ahmed N. Hegazy, Andreas Bergthaler

**Affiliations:** 1 Institute of Hygiene and Applied Immunology, Department of Pathophysiology, Infectiology and Immunology, Medical University of Vienna, Vienna, 1090 Austria; 2 CeMM Research Center for Molecular Medicine of the Austrian Academy of Sciences, Vienna, 1090 Austria; 3 Charité – Universitätsmedizin Berlin, corporate member of Freie Universität Berlin and Humboldt-Universität zu Berlin, Department of Gastroenterology, Infectious Diseases and Rheumatology, 12203 Berlin, Germany; 4 Deutsches Rheuma-Forschungszentrum, ein Institut der Leibniz-Gemeinschaft, 10117 Berlin, Germany

**Keywords:** Lymphocyte activation, Immunological surveillance

CD8^+^ T cell exhaustion is a phenomenon often observed in individuals battling chronic diseases like cancer and chronic viral infections. Prolonged exposure to antigens desensitizes CD8^+^ T cells, leading to a diminished capacity to mount effector responses. This state of exhaustion is characterized by decreased expression and secretion of cytolytic proteins and cytokines, coupled with upregulation of inhibitory receptors (e.g., PD-1, TIM-3, LAG-3) [1]. T cell exhaustion unfolds along a trajectory where progenitor exhausted CD8^+^ T cells (T_PEX_) give rise to a larger number of terminally exhausted CD8^+^ T cells (T_EX_) [2]. Modern cancer therapy aims to re-invigorate pre-existing exhausted T cells by blocking its inhibitory receptors, which is known as immune checkpoint inhibition (ICI) therapy. T_PEX_ are most amendable to ICI therapy, which then give rise to a substantial burst of functional CD8^+^ T cells and thus driving anti-tumor responses [3]. However, not all patients respond to ICI therapy and thus creating a further need to identify crucial factors that drive CD8^+^ T cell exhaustion to enhance the efficacy ICI therapy.

Over the past decade, it has become apparent that immunometabolic plasticity is central for the control of T cell responses during immune challenges [4]. During chronic infections, a bioenergetic deficit in CD8^+^ T cells, likely triggered through the early reduction of both glycolytic and oxidative phosphorylation (OXPHOS)-dependent pathways, even proceeds their reduced functionality [5]. Several studies over the past years have highlighted that an impaired mitochondrial state marked by increased production of reactive oxygen species (ROS) is a key feature of T cell exhaustion [6–8]. While T_PEX_ exhibit a mitochondrial state with high respiratory capacity, this progressively deteriorates in T_EX_ [9]. In line with this, CD8^+^ T cells with depolarized mitochondria were non-proliferative and hypofunctional, while expressing increased exhaustion markers [8]. Promoting mitochondrial quality control by induction of mitophagy improved mitochondrial fitness and thus reduced CD8^+^ T cell exhaustion [8]. This was linked to an attenuated intracellular production of mitochondrial ROS. Conversely, reducing ROS through antioxidant treatment could rescue CD8^+^ T cell functionality and proliferation [6]. While this collective evidence strongly suggests that mitochondrial dysfunction is a main driver of CD8^+^ T cell exhaustion, formal proof was still lacking.

In this study, which assessed as preprint and has in the meantime been published, Wu et al. [10] dissected the metabolic requirements that drive CD8^+^ T cell exhaustion. In line with previous reports the authors found that T_PEX_ relied on mitochondrial respiration, while T_EX_ reduced OXPHOS gene expression and shifted their metabolism towards glycolysis. Using a photoconvertible fluorophore located in the mitochondria, the authors found that T_PEX_ have a higher mitochondrial turnover than T_EX_, pointing towards a dysfunctional mitochondrial quality control in T_EX_. This was previously associated to a reduced mitophagic flux leading to the accumulation of mitochondria in exhausted tumor-infiltrating lymphocytes [8]. Overall, the data follows previous findings of a higher dependency of T_PEX_ on mitochondrial respiration compared to T_EX_.

To causally link mitochondrial respiration with the development of CD8^+^ T cell exhaustion, the authors genetically deleted the mitochondrial inorganic phosphate transporter *Slc25a3* (also known as mPiC) in T cells. This abrogated the influx of inorganic phosphates into the mitochondrial lumen and thus prevents the conversion of ADP to ATP. Expectedly, loss of this transporter reduced basal mitochondrial respiration, while it increased basal glycolysis in CD8^+^ T cells as measured by metabolic flux and heavy carbon-labelled glucose tracing. Moreover, deletion of *Slc25a3* was sufficient to drive increased expression of exhaustion-associated markers (e.g., TIM-3, LAG-3), but also reduced their ability to produce multiple cytokines upon restimulation. Moreover, *Slc25a3*-KO cells exhibited an in increased propensity to undergo apoptosis upon activation. Direct comparison of WT vs. *Slc25a3*-deleted antigen-specific T cells in the same chronically-infected host revealed that mitochondrial dysfunction induced by *Slc25a3* deletion substantially increased T cell exhaustion. Consequently, WT cells eventually outcompeted *Slc25a3*-deficient CD8^+^ T cells. Remarkably, *Slc25a3*-deficient T cells also showed an increased exhaustion-associated phenotype in acute viral infections with LCMV Armstrong, which was absent in WT mice. This suggests that mitochondrial impairment can drive exhaustion-like programs independent of antigen persistence. To validate the driving role of mitochondrial respiration on T cell exhaustion, the authors overexpressed *Slc25a3* in antigen-specific T cells, which boosted mitochondrial respiration and shifted CD8^+^ cells towards an T_PEX_ phenotype. Collectively, the authors revealed an intriguing direct genetic link of mitochondrial dysfunction to T cell exhaustion, thus suggesting that mitochondrial dysfunction is not a mere bystander, but rather driver of CD8^+^ T cell exhaustion. However, the relevance of the model to study T cell exhaustion more broadly may be restricted though the increased tendency of *Slc25a3*-deficient T cells to undergo exhaustion-associated apoptosis.

Finally, the authors focused to identify the molecular mechanism that links mitochondrial dysfunction to the expression of exhaustion-associated markers. For this, the authors first compared the metabolome of *Slc25a3*-deficient CD8^+^ T cells with WT CD8^+^ T cells and found a reduced presence of metabolites of the pentose phosphate pathway (PPP) and a reduced availability of NADPH. NADPH is an important antioxidant generated from the PPP, suggesting that terminal differentiation may be associated with increased oxidative stress. In line with this hypothesis, the authors found that *Slc25a3*-deficient T cells have increased mitochondrial stress (i.e., ROS). Treating these cells with the antioxidant N-acetyl cystine (NAC) reduced their elevated exhaustion marker expression to WT levels, suggesting that mitochondrial respiration is upstream of CD8^+^ T cell exhaustion marker expression. As such, Slc25a3-deficient T cells mimic aberrant oxidative stress responses observed in exhausted T cells [6].

ROS has far-reaching implications for cellular functions and can alter transcription via multiple pathways. As transcription factors NFAT and HIF1α can both be controlled by ROS, the authors tested their impact on exhaustion marker expression in the *Slc25a3*-deficient mouse model. Remarkably, in contrast to previous reports NFAT was dispensable for exhaustion marker expression [6], instead ROS-dependent HIF1α stabilization drove the transcriptional control of many exhaustion-associated marker genes in *Slc25a3*-deficient T cells. Interestingly, these findings contrast previous reports suggesting that, under hypoxic conditions, CD8^+^ T cell exhaustion is driven in a HIF1α-independent and NFAT-dependent manner [7]. Since *Slc25a3*-deficient T cells become increasingly more exhausted even in the absence of exogenous stress such as hypoxia, this suggests that defective mitochondrial respiration on its own acts as a cell-autonomous trigger of CD8^+^ T cell exhaustion.

To validate the role of HIF1α in CD8^+^ T cell exhaustion, the authors deleted *Hif1a* from T cells and found that it expectedly reduced glycolysis and shifted their phenotype towards T_PEX_ characteristics by reducing the expression of terminal exhaustion markers. This finding implicated HIF1α activity in the differentiation of terminally exhausted CD8^+^ T cells. Lastly, pharmacological modulation of CD8^+^ T cell metabolism by pre-treating antigen-specific CD8^+^ T cells with the glucose analogue 2-Deoxyglucose (2-DG), which inhibits glycolysis, improved CD8^+^ T cell function upon chronic viral infection. Similarly, pre-treatment of CAR T cells with 2-DG slightly improved their anti-tumor response. Together, this data suggests that metabolic rewiring of T cells could prevent induction of T cell exhaustion, although more research is needed to delve into the epigenetic modification induced by targeting immune cell metabolism.

**Fig. 1:**
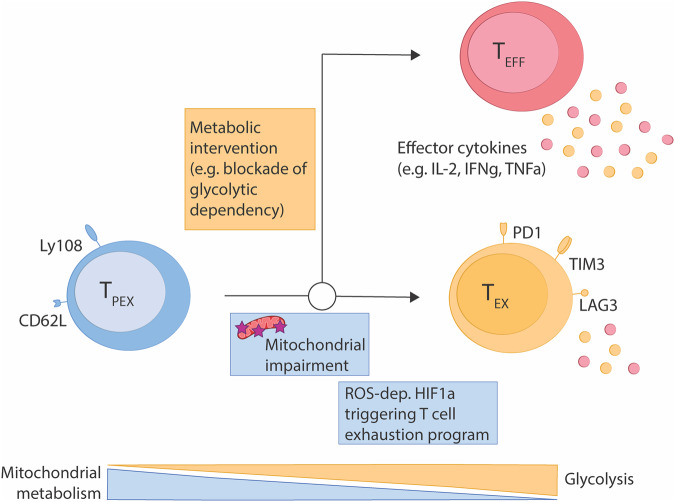
Genetic disruption of mitochondrial metabolism drives ROS-HIF1α dependent exhaustion programs in CD8^+^ T cells. Impairment of mitochondrial fitness represents a key infliction point of CD8^+^ T cell exhaustion in response to viral chronic infection. This implicates drugs targeting CD8^+^ T cell metabolism as an important new avenue to increase immune checkpoint inhibition and CAR T cell therapies.

This study provides an important causative link between mitochondrial quality, mitochondrial respiration and T cell exhaustion (Fig. 1). While these findings are exciting, the effects of ICI therapy on re-wiring T_PEX_ metabolism continues to be ill-defined. Understanding the metabolic control of T cell re-invigoration feels like the logical next step to develop therapeutic approaches that target the immunometabolic programs of T_PEX_. Identification of molecular and metabolic factors that control terminal exhaustion have the potential to complement classical ICI therapy and improve efficiencies and durability of ICI therapies in the future.
